# Integrating simulation into surgical training: a qualitative case study of a national programme

**DOI:** 10.1186/s41077-023-00259-y

**Published:** 2023-08-18

**Authors:** Adarsh P. Shah, Jennifer Cleland, Lorraine Hawick, Kim A. Walker, Kenneth G. Walker

**Affiliations:** 1 Centre for Healthcare Education Research and Innovation (CHERI), University of Aberdeen, Aberdeen, UK; 2 Lee Kong Chian School of Medicine, Nanyang Technological University, Singapore, Singapore; 3 NHS Education for Scotland, Centre for Health Science, Old Perth Road, Inverness, IV2 3JH UK

**Keywords:** Simulation-based education, Surgical training, Programme evaluation, Implementation science, Qualitative research, Case study, Normalisation process theory

## Abstract

**Background:**

Applying simulation-based education (SBE) into surgical curricula is challenging and exacerbated by the absence of guidance on implementation processes. Empirical studies evaluating implementation of SBE interventions focus primarily on outcomes. However, understanding the processes involved in organising, planning, and delivering SBE adds knowledge on how best to develop, implement, and sustain surgical SBE. This study used a reform of early years surgical training to explore the implementation of a new SBE programme in Scotland. It aimed to understand the processes that are involved in the relative success (or failure) when implementing surgical SBE interventions.

**Methods:**

This qualitative case study, underpinned by social constructionism, used publicly available documents and the relevant surgical SBE literature to inform the research focus and contextualise data obtained from semi-structured interviews with core surgical trainees (*n* = 46), consultant surgeons (*n* = 25), and key leaders with roles in surgical training governance in Scotland (*n* = 7). Initial data coding and analysis were inductive. Secondary data analysis was then undertaken using Normalisation Process Theory (NPT). NPTs’ four constructs (coherence, cognitive participation, collective action, reflexive monitoring) provided an explanatory framework for scrutinising how interventions are implemented, embedded, and integrated into practice, i.e. the “normalisation” process.

**Results:**

Distributed leadership (individual SBE initiatives assigned to faculty but overall programme overseen by a single leader) and the quality improvement practise of iterative refinement were identified as key novel processes promoting successful normalisation of the new SBE programme. Other processes widely described in the literature were also identified: stakeholder collaboration, personal contacts/relational processes, effective communication, faculty development, effective leadership, and tight programme management. The study also identified that learners valued SBE activities in group- or team-based social environments over isolated deliberate practice.

**Conclusions:**

SBE is most effective when designed as a comprehensive programme aligned to the curriculum. Programmes incorporating both group-based and isolated SBE activities promote deliberate practice. Distributed leadership amongst faculty attracts wide engagement integral to SBE programme implementation, while iterative programme refinement through regular evaluation and action on feedback encourages integration into practice. The knowledge contributed by critically analysing SBE programme implementation processes can support development of much needed guidance in this area.

**Supplementary Information:**

The online version contains supplementary material available at 10.1186/s41077-023-00259-y.

## Background

Simulation-based education (SBE) plays an increasingly evidence-based role in surgical training [1–7]. SBE is considered a powerful adjunct to clinical workplace training [8], particularly in this era of diminishing time for on-the-job training, increasing patient case complexity, technological advances in surgery, and increased focus on patient safety [5, 9–11]. However, effective implementation and integration of SBE initiatives into wider surgical curricula have encountered significant challenges [12, 13]. This is perhaps unsurprising; SBE is a complex intervention involving multiple behavioural, technological, and organisational components [14] with predictable barriers to change at multiple levels (e.g. learner [15], faculty [16], and systems [17]).

Despite this, empirical studies evaluating the implementation of SBE interventions tend to report more on outcomes [3, 18–21] than on descriptions or critical analysis of the processes involved in organising, planning, and delivering SBE. Of course, there are some exceptions [22–25], but generally, the implementation of surgical SBE is an under-researched area.

Given that surgical SBE is costly and resource intensive [9, 26, 27], understanding how to effectively develop, implement, and sustain SBE interventions is of interest and importance to all stakeholders. To address this gap in the literature, we used a curriculum reform of early years surgical training as the ideal opportunity to explore the implementation of a new SBE programme. Our research question was as follows: what processes promoted or inhibited the implementation of a comprehensive programme of surgical SBE (henceforth called “Programmatic SBE”)? Using a case study approach, our aim was to understand the processes that are involved in the relative success, or failure, when implementing surgical SBE interventions.

## Methods

### Context

In the UK, Core Surgical Training (CST) is a 2-year, broad-based training programme which follows medical school and generic foundation training (internship) and is a requisite for higher surgical specialty training (residency). Our specific context is Scotland, one of the UK’s four countries, where there are two CST programmes (East and West of Scotland), each with a Training Programme Director. Forty to fifty trainees are enrolled annually across CST in Scotland. They train in a range of hospitals including rural general, district general, and urban tertiary hospitals.

NHS Education for Scotland (NES) is responsible for the delivery and governance of CST. Direct oversight of CST is by the Surgical Specialties Training Board (SSTB), the Scottish equivalent of Schools of Surgery in England. The strategy for SBE within CST was designed by the Scottish Surgical Simulation Collaborative (SSSC). However, the surgical training landscape is complex and includes other players such as the Surgical Royal Colleges [22, 28]. We come back to this later.

From 2018, a major reform of CST across the UK, “Improving Surgical Training” (IST), was piloted to improve surgical trainees’ experience and to ensure “the product at the end of training meets current and future patient needs” [29] (p2). IST in Scotland included all CST posts and, after 3 years of pilot, became “business as usual”, i.e. recommendations for improving surgical education and training were incorporated into routine CST.

**Fig. 1:**
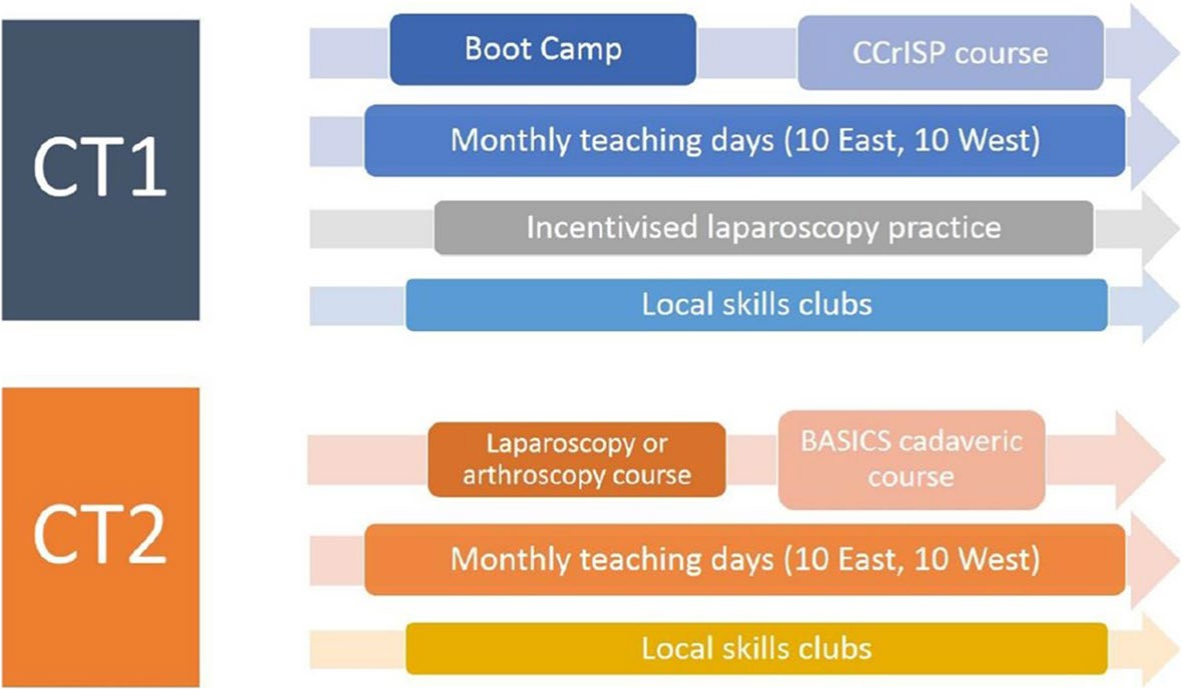
Summary of Programmatic SBE implemented across the two core surgical training programmes in 2018

A key recommendation of IST was that “simulation should be embedded and enhanced within surgical curricula,” and that “technical and non-technical skills are taught and developed in a simulated environment” [29] (p3). In Scotland, this resonated with existing plans to develop a “Simulation Strategy” (Fig. 1) (NES, 2021; Walker and Shah, 2021), a comprehensive SBE programme aligned to the CST curriculum (Intercollegiate Surgical Curriculum Project, 2021). All components of this programme were managed centrally with the exception of the “skills clubs” — a trainee-led, near-peer initiative delivered and managed at the individual departmental level. The focus of our study is the implementation of the Programmatic SBE as a whole.

### Methodology

This research is nested in a wider qualitative case study evaluating the Improving Surgical Training (IST) curriculum reform [28, 30, 31]. Both this study and the wider case study were underpinned by social constructionism, acknowledging that reality is produced through the interchanges between people and objects and shared activities, with knowledge and the individual embedded in history, context, culture, language, and experience [32]. This position aligns with Merriam’s [33] approach to case study methodology.

We drew on publicly available documents [29] to help orientate us to the IST recommendations and the relevant literature [15, 22, 34–36] to understand the existing landscape of surgical SBE in Scotland. Both sources of data also helped to inform the research focus and to contextualise the interview data. We then used semi-structured interviews of Core Trainees (CTs), consultant surgeons (trainers), and key leaders, i.e. those with roles in surgical training governance within NES, the two Scottish Surgical Royal Colleges, and the SSSC in Scotland. Interviews explored participants’ views regarding the processes of developing and implementing Programmatic SBE in the context of a curriculum reform of early years’ surgical training.

### Participants

Recruitment of prospective participants was conducted via emails. The training programme directors emailed invitations to CTs (*n* = 91) and trainers (*n* = 70) on our behalf between April 2020 and August 2020 (CTs and trainers) and February 2021 and May 2021 (trainers only). We also wanted the views of key leaders (*n* = 7) — people who had specific knowledge and understanding of the IST reform plan and implementation strategy for both IST and Programmatic SBE. Key leaders were identified by the team and then e-mailed directly by the lead researcher (APS). We requested all research participants to assist us in snowball sampling [37]. Positive responses to our introductory e-mails were followed up with an e-mail providing more information about the study and data collection.

### Data collection

We developed a semi-structured interview schedule [38] informed by the IST document [29], associated publications [39, 40], and the literature pertaining to surgical SBE in Scotland [5, 15, 22, 34–36, 41]. Interview questions were designed to explore participants’ perceptions and experiences of Programmatic SBE. The questions focussed on exploring the development and implementation of Programmatic SBE, its alignment with the CST curriculum, and the perceived value, affordances, and limitations of Programmatic SBE. After obtaining written consent from participants, APS conducted all interviews virtually via the Microsoft Teams platform.

### Data analysis

Interviews were digitally audio-recorded for later transcription, during which participants were anonymised. Transcripts were entered into the qualitative data analysis software NVivo v12.0 (QRS International Pty Ltd., Doncaster, Victoria, Australia) to facilitate data management and coding. We conducted a thematic analysis to identify themes and subthemes [42]. After team discussions of preliminary codes and resolution of any coding disagreements, coding occurred iteratively and inductively, focusing throughout on the research question. Data sufficiency was indicated when representation from all CST training sites and surgical specialties was achieved and thematic saturation noted [43].

**Fig. 2:**
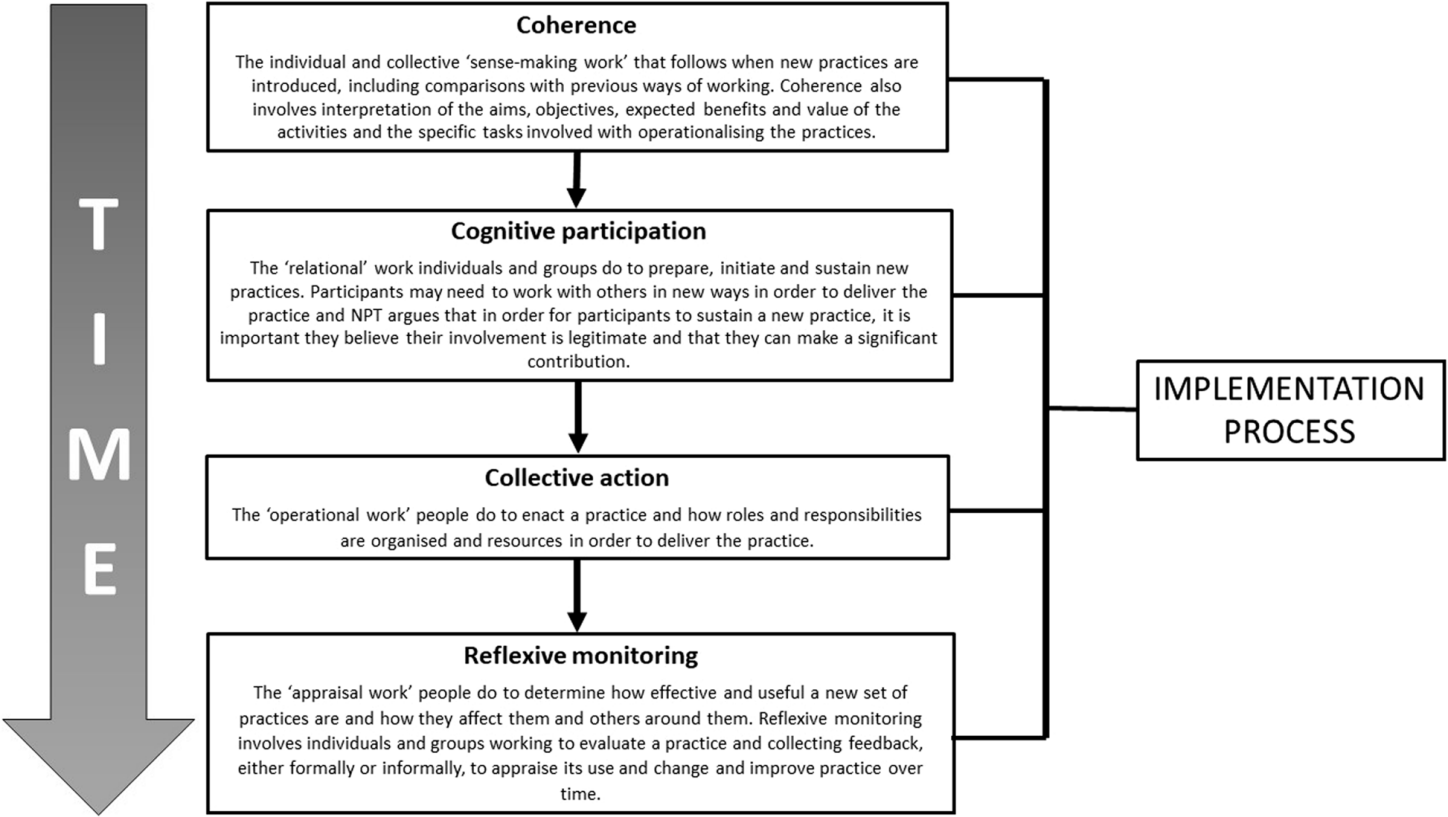
The four constructs comprising Normalisation Process Theory (adapted from May et al. [46]) and the respective definitions for each construct [24]

Following this and after further team discussions, we drew on Normalisation Process Theory (NPT) to identify and understand the process(es) enabling or inhibiting successful embedding and integration of Programmatic SBE within CST. NPT provides an explanatory framework for scrutinising how interventions or innovations are implemented, embedded, and integrated into practice [44, 45]. NPT has been widely used in process evaluations of complex interventions in healthcare [46], health professions education [24], and medical practice [47]. NPT proposes that normalisation has four generative constructs: coherence, cognitive participation, collective action, and reflexive monitoring (Fig. 2) [44, 48].

These four NPT constructs can be used to understand sense-making amongst individuals and groups via the perceived aims, objectives, and value of the intervention or new practice (coherence), the commitment of individuals and groups to implement and sustain the intervention (cognitive participation), individual and group engagement through the work they do and using the resources available to deliver the intervention (collective action), and individual and group experiences of how the new practice affects them and others (reflexive monitoring) [24, 45, 49]. Although presented linearly (see Fig. 2), there is an assumption of overlap and concurrent actions in an implementation process [46].

### Reflexivity and rigour

We considered our positions and relationships with the data continually and critically in view of our different interdisciplinary backgrounds (surgery, psychology, pharmacology, and nursing) and different levels of knowledge and experience of surgical simulation, education, training, and research [50, 51]. For example, as a surgical trainee from another UK country, AS was both an insider and an outsider — external to Scotland’s CST programme but an insider as a surgical trainee who had knowledge of the structures and systems within UK surgical training. We acknowledge that his positionality may have influenced data collection and analysis. However, to mitigate this, the wider multidisciplinary research team supported AS during data collection and analysis, encouraging considerations of the different perspectives on the data, keeping his “eyes open”, and avoiding making assumptions [52].

## Results

Forty-six trainees, twenty-five trainers, and seven key leaders responded to the email invitations. Trainees and trainers worked in 27 different hospitals, representing all surgical specialties except neurosurgery, and 13 of the 14 territorial health boards in Scotland providing Core Surgical Training. The mean interview duration was 48 min. All transcripts were utilised in the analysis.

There are different ways of presenting qualitative data; the findings in this study are presented as themes corresponding to the four constructs of Normalisation Process Theory. These are discussed separately in the following section. Participants have been anonymised and identified as trainee (CT), trainer (TR), or key leader (KL). We report verbatim quotes to aid confirmation of findings and to help the reader follow the logic of the story. An ellipsis (…) indicates where text that has been cut for brevity.

### Coherence: the value of programmatic SBE

NPT posits that successful implementation of a new practice or intervention requires participants’ understanding of what it is and what it entails and acceptance of its aims and objectives. Participants may draw upon comparisons with existing practices, or the status quo, to help with this sense making. This was apparent in our data.

Originally, surgical simulation opportunities were unevenly distributed across the country in an organic rather than strategic manner with “pockets of great practice and expertise and plenty of facility, but little in the way of strategy… things were happening ad hoc” (KL06). For example, a surgical bootcamp that initially started in 2009 as a local initiative was later integrated into CST in 2012 [22, 35]. In 2014, a take-home laparoscopic simulator initiative was introduced, but initial engagement from CTs was poor, instigating research and development to ensure that this initiative was (retrospectively) “very, very carefully worked out and developed over a number of years” (KL06) [15, 22, 34, 36]. Simulation training days “were less structured [and] there was a lot less simulation content” (KL01).

**Fig. 3:**
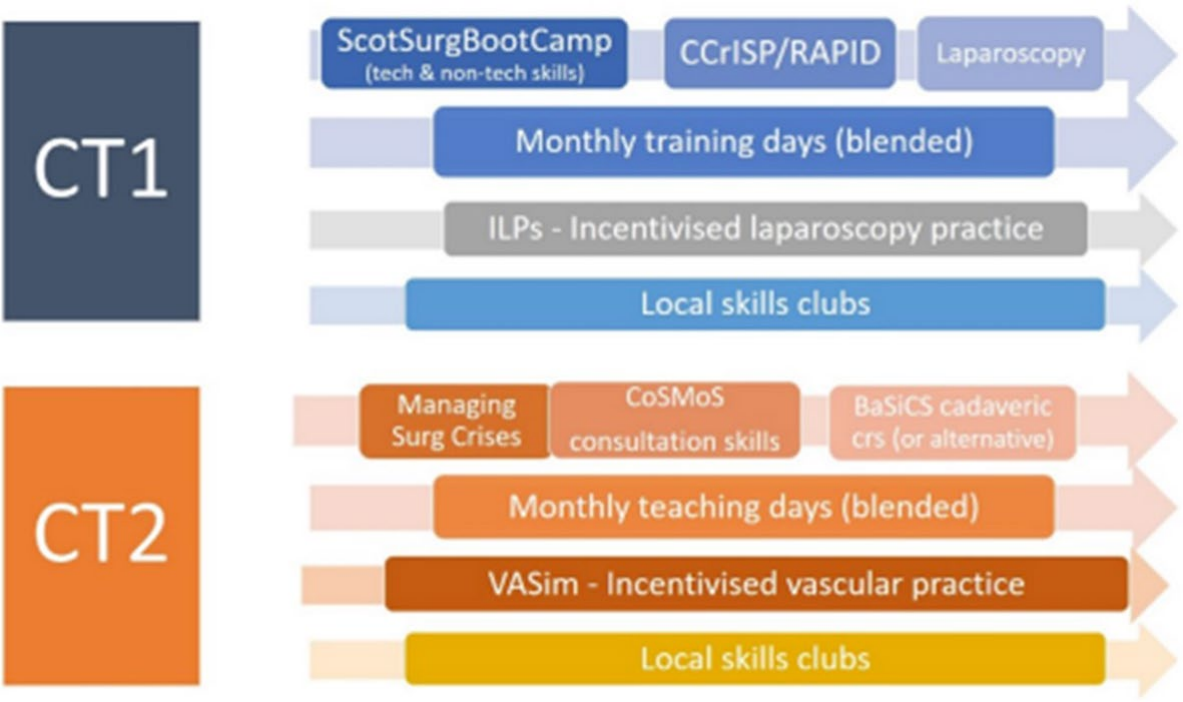
The updated Programmatic SBE implemented from 2021 onwards following continuous evaluation and iterative refinement

In view of this, “a more structured approach to delivery” (KL04) was needed. The “Simulation Strategy” (i.e. Programmatic SBE) brought together several independent initiatives into one comprehensive programme “mapped to the curriculum and mapped to the resource and opportunity” (KL06). An overview is presented in Fig. 1 and the later revised version in Fig. 3. Programmatic SBE was designed as “an adjunct to clinical training” [5], envisaged to deliver a better training experience. This was seen as bringing benefit the following:

"[by] focusing on a lot of simulation in advance of getting into theatre helps them [trainees] understand the basic principles... which makes their learning experience in theatre so much more valuable" (TR07).

Trainers also believed that the inclusion of nontechnical skills was a valuable component of Programmatic SBE: “the personal skills and communication that need to be emphasised—that’s more important than any of the clinical training” (TR12).

### Cognitive participation: commitment to programmatic SBE implementation

This construct refers to obtaining participant commitment to initiate implementation and depends upon buy-in from participants arising from shared beliefs about the reform [44].

The 2015 IST proposal aligned with Scotland’s existing plans to develop and implement Programmatic SBE. IST provided a catalyst or enabler, triggering “buy-in from the Deanery (NES) [and] from the Scottish Government” (KL03). However, “buy-in” does not lead linearly to implementation, particularly in complex settings. Commitment from different groups was necessary; some key stakeholders had to be brought on board first and some key developments put in place in order to then engage others.

First, it was the formation, in 2016, of “a small [IST] working group of, at most, maybe a dozen members” (KL01) who were responsible for steering IST and Programmatic SBE implementation across both CST programmes. There was an established chain of command, with the IST working group accountable to the Surgical Specialties Training Board (SSTB), which in turn reported to the NES Executive Board. An experienced SSTB member led the IST working group, whose members were consultant surgeons with expertise in surgical education and experience of delivering training within Scotland. Some members also had roles in other groups such as the Scottish Surgical Simulation Collaborative (SSSC), which had input into Programmatic SBE development, and the Surgical Royal Colleges. The multiple roles of the IST working group members promoted development of networks with other CST stakeholder groups.

Second, the role of “IST Simulation Lead” was created in 2016. Enabled by membership of both the IST working group and the SSSC, the IST Simulation Lead was expected to “put together the structured [SBE] programme and the elements of it, building on what was there before” (KL04) and get the draft proposal for Programmatic SBE “approved by the SSTB” (KL06), following which it was presented “to Scottish Government to pitch for funding” (KL06):

"…our proposal [for Programmatic SBE] was that it should be for the entire CST programme and after a few discussions with Government officials, they were very interested" (KL04).

Relationships were critical in facilitating positive dialogue and negotiation between those at higher levels within NES and government:

"The chair of the Shape of Training Implementation Group is XX, who happens to work as a medical advisor for the Scottish government… he’s a surgeon" (KL01).

With funding secured, focus turned to increasing the engagement and buy-in from those enacting Programmatic SBE in the healthcare context: the healthcare organisations and consultant surgeons. Participants emphasised the importance of “winning the hearts and minds of the DMEs [Directors of Medical Education]” (KL04) within the higher management structures of healthcare organisations. Thus, in 2017 (the year before IST implementation), a senior member of the IST working group “met with all the DMEs in Scotland” (KL01) and went round “the Health Boards talking to the DMEs, getting that buy-in” (KL04).

NES organised “trainer bootcamps” and workshops for consultant surgeons with supervisor/trainer responsibilities. Details of Programmatic SBE’s components and how they work were formally communicated at these events:

"We had 2 trainer bootcamps; we had a 2-day one for the first year, and then the second year we had a 1-day one; and they were pretty well received. We also had a 1-day workshop [where] in the morning we’d run a simulation training session for trainers and then in the afternoon, we had a sort of feedback [session]" (KL01).

These faculty development initiatives legitimised trainer involvement in the implementation process. As one participant noted, trainers were “so enthused and excited about this new [SBE] programme [because] they haven’t seen it before in any discipline, never mind surgery” (KL04).

In short, Programmatic SBE implementation was promoted by the collective work of individuals and stakeholder groups to drive buy-in and engagement through a web of existing and new collaborative networks.

### Collective action: operationalising programmatic SBE

This construct refers to the roles and responsibilities undertaken by individuals and groups and how resources are organised to enact the intervention into practice. There is clearly an overlap between this construct and that of cognitive participation. Just as the previous section, ownership and communication were critical in winning the hearts and minds of key stakeholders such as Scottish Government or consultant surgeons. At the next stage in the process, operationalisation “on the ground”, strategies had to be found to ensure ongoing engagement from the surgical community, and to overcome the practical issue of trainees being spread across a large geographical area. While the IST Simulation Lead had oversight of all components within Programmatic SBE and orchestrated the administrative and governance tasks:

"I have all my time consumed by the huge logistics of coordinating so many events or so many faculty" (KL06).

Faculty and events were distributed across five major Scottish cities (Edinburgh, Glasgow, Dundee, Aberdeen, and Inverness). A key process in overcoming this challenge was distributed leadership, i.e. dedicated faculty was assigned responsibility for each SBE component based on their location and/or institution:

"A number of the faculty really took the challenge and ran with it well, and so that made a difference. It wasn’t my job to take over the monthly training days, and it was good that we had a deputy TPD and a TPD on each side of the country doing that" (KL06).

Other examples include delivery of the cadaveric and non-cadaveric technical and non-technical skills courses in collaboration with the two Scottish Surgical Royal Colleges (Glasgow and Edinburgh). The residential surgical bootcamp stayed where it was, in Inverness, Scotland [22, 35], but was co-delivered by faculty travelling in from across Scotland. The skills clubs [15] were the responsibility of individual surgical departments/healthcare institutions and run by CTs themselves, with the assistance of senior surgical trainees (registrars). Managing the different components of Programmatic SBE in this way conferred a sense of ownership across the wide surgical community:

"You know to have a team that shares the same vision – the faculty we have around bootcamp, the faculty around the other courses – [is] very, very encouraging to work with" (KL06).

### Reflexive monitoring: experiences of programmatic SBE

This construct refers to appraising individual and group subjective experiences of the new practice (in this case, Programmatic SBE) and learning from ongoing experiences to adapt it (Fig. 3). This was apparent at various levels. At the level of trainee and trainer, the engagement and feedback were positive. For example, trainers reported seeing trainees progress because of programmatic SBE:

"You do the see the difference when they come [having practised] they are much more confident… and you can actually tell people have or who haven’t practised" (TR19).

Trainers used their knowledge of Programmatic SBE when giving feedback in the workplace. For example, one participant described an instance when they received a specific instruction similar to a simulated laparoscopic task so as to improve their technical ability:

"My consultant would talk about having more tension in the left than the right [hand]. She knew the things that were in the [laparoscopic] box, so she’d be like, “it’s like when you do that [manoeuvre]" (CT04).

CTs reported positive experiences associated with Programmatic SBE engineering a healthy sense of competition and peer support. In respect to the first of these, an award of “a certificate or medal if you do the best out of the cohort [in] the elite tasks” (CT06) led to related goal-setting behaviours from a trainee: “I gave myself a task of getting an A in each [skill] so that I would practise the skills a bit more” (CT06). The social gains were very apparent. For example, with respect to the skills clubs, the availability of near-peer support ensured there was “always someone to help you with things if you find it tricky” (CT21). The residential, 4-day bootcamp offered CTs multiple opportunities to interact with faculty and peers, thereby fostering a sense of a shared purpose: “you meet colleagues for life, and you make friends for life as well, so there’s a lot of collegiality” (CT12). These positive experiences seemed to encourage CTs to engage with the SBE activities more widely: “it’s nice having someone there who you can talk to [be they fellow trainee or faculty] … it’s easier to be engaged with it [Programmatic SBE]” (CT41).

The feedback was also positive at a more systems level: Programmatic SBE development appeared to have addressed the challenges with recruitment (“our recruitment’s gone from 50% at ST3 in general surgery to 100% for the last 3 years” (KL01)) and improved overall quality of the training experience:

"… speaking to a number of the trainees involved in the [CST] programme, and also trainers, it’s been very positive. There’s better focus in terms of the regional teaching, they [trainees] have all thoroughly appreciated the sort of structured elements to the 2 year programme, particularly around simulation … from the ARCP [Annual Review of Competency Progression] outcomes, on feedback from the bootcamps, on exam results, it all seems to be we’re getting to a much better place than we would have been a few years ago" (KL04).

## Discussion

To the best of our knowledge, this is the first study in the surgical education literature to explore the implementation of a comprehensive programme of SBE.

Our findings add to the simulation literature by examining the process(es) by which Programmatic SBE was implemented, embedded, and integrated into surgical education and training. Context — particularly the physical (geography of surgical SBE), practical (historical nature of surgical SBE and people involved), and social (beliefs and values of participants) — influenced the sensemaking process. The work of individuals (e.g. the IST Simulation Lead) and groups (e.g. the IST Working Group) was foregrounded by the institutional context (the multiple stakeholder groups) and their networks, thereby promoting programmatic SBE implementation. Processes such as distributed leadership afforded wide engagement and ownership amongst faculty. Appealing to trainees’ competitive spirit seemed to help engagement, but the social aspect of programmatic SBE activities was most important to learners.

The processes identified in our data concur with findings from other studies evaluating curriculum reforms with respect to stakeholder collaboration [2, 53–55], personal contacts/relational processes [54], communication [54], faculty development [54], and effective leadership and tight programme management [53, 56]. However, the processes of distributed leadership (and the resultant wide engagement and sense of ownership) and the quality improvement practice of iterative refinement to integrate and sustain programmatic SBE are seldom described in curriculum reform [57] or surgical education and training [58].

### Implications for policy, practice, and research

The subjective experiences described in this study reinforce the value of encasing SBE activities within a programme rather than standalone initiatives. SBE programmes should be thought of as an iterative, quality improvement process that is constantly evaluated to enable adaptation to the needs of stakeholders and/or the curriculum. Thus, based on our findings, we recommend that those currently involved in developing and/or implementing SBE interventions establish a receptive, committed group of faculty who are empowered by distributed leadership and engage in reflexive monitoring.

A significant finding within our data — that programmatic SBE fostered learning environments facilitating collegiality, networking, goal directedness, and social affiliation — contrasts with the isolated nature of deliberate practice from take-home simulators, which proliferated during the peak of the COVID-19 pandemic [36, 59–62]. While deliberate practice with take-home simulation may facilitate technical skills acquisition [63], it denies learners the opportunities for “informal” learning offered within social, team-based environments, as was the case with programmatic SBE. Given the appetite for team-based SBE demonstrated by our participants, we encourage programme directors to evaluate and formulate programmatic SBE with initiatives that promote deliberate practice in groups where possible [63, 64].

Our study focused on the process of implementation. Key stakeholders will also be interested in outcomes and costs. Though the SSSC estimated that Programmatic SBE would pay for itself (a cost to Scottish Government of £2000 per trainee per year) in savings in operating room time, improved outcomes, and reduced litigation, it remains the case that the economics and the Kirkpatrick level 3 and 4 outcomes for this type of simulation remain under-researched [65, 66].

This study describes the processes identified in one context. Further research using NPT or alternative theoretical frameworks to explore SBE programme implementation in other contexts may well identify similar or different processes. Such empirical work may enable development of best evidence guidance with regard to SBE implementation.

### Strengths and weaknesses

A major strength of this study lies in gathering the views of different stakeholders. Most studies looking at the implementation of SBE tend to explore the views of one group only (e.g. Karam et al. [18], Nousiainen et al. [20], Davis et al. [21], Chang et al. [67]), whereas we intentionally recruited participants from three different, relevant groups of stakeholders to obtain a range of perspectives [68].

Our use of theory is also a strength, particularly given SBE research has been criticised for its paucity of theory [69]. NPT originates from the field of implementation science and has its roots in medical sociology [14]. We carefully considered this theory and believed its assumptions were congruent with our approach and question [70]. The NPT coding manual assisted with organising the data at the point of secondary analysis [48], helping us examine engagement with SBE at a social process level, a new perspective on a phenomenon which is usually scrutinised using cognitive learning theories (e.g. instructional design [71, 72], deliberate practice [73–75]). However, as noted in our study, implementation processes, like social processes, are complex, dynamic, and emergent such that any or all of the four NPT constructs can occur concurrently. Thus, NPT cannot ascertain causation between the enabling or inhibiting factors and outcomes of change processes, thereby limiting its power to predict outcomes [44]. The transferability of NPT in explaining how Programmatic SBE was normalised remains to be tested in other contexts. Finally, NPT has been criticised because it provides retrospective explanations of social relationships or processes of the studied phenomenon (May and Finch, 2009). We were looking for an understanding of processes which happened over time so considered this a strength rather than a limitation.

## Conclusion

Surgical SBE is most effectively delivered as a comprehensive programme of initiatives aligned to a curriculum rather than standalone initiatives. Achieving normalisation of surgical SBE programmes (or interventions/innovations) requires an understanding of the processes that enable or inhibit the various stages of implementation. Engaging in critical analysis in addition to evaluating outcomes (be that trainee performance, patient, or health economics) will develop knowledge that may initiate the development of much needed guidance on how best to implement surgical SBE programmes, interventions, or innovations.

### Supplementary Information

**Additional file 1:** **Supplementary Table 1.** Illustration of how the coding framework mapped to the four NPT constructs. How the codes, sub-themes, and themes were organised during initial thematic analysis, and how they were mapped to the four NPT constructs following secondary data analysis.

## Data Availability

The datasets used and/or analysed in this study are available from the corresponding author on reasonable request.

## Abbreviations

CST: Core surgical training
DME: Director(s) of medical education
IST: Improving surgical training
NES: NHS Education for Scotland
NPT: Normalisation Process Theory
SBE: Simulation-based education
SSSC: Scottish Surgical Simulation Collaborative
SSTB: Surgical Specialties Training Board
