# Supplementary material for: Integrating simulation into surgical training: a qualitative case study of a national programme
Source: Adv Simul (Lond). 2023 Aug 18;8:20. doi: 10.1186/s41077-023-00259-y (PMC10436455; doi:10.1186/s41077-023-00259-y)
Supplement: Supplementary file 1 — Additional file 1: Supplementary Table 1. Illustration of how the coding framework mapped to the four NPT constructs. How the codes, sub-themes, and themes were organised during initial thematic analysis, and how they were mapped to the four NPT constructs following secondary data analysis. [file 41077_2023_259_MOESM1_ESM.docx]

| CODE | SUB-THEME | THEME | NPT CONSTRUCT |
| --- | --- | --- | --- |

| - Delivering on existing quality indicators - Improve recruitment to surgery - Embed simulation into training - Parity at higher specialty training - Equality across both programmes | Core Surgical Training programme level aims | Aims and objectives of programmatic SBE | COHERENCE |
| --- | --- | --- | --- |
| - Improve overall training experience - Remedy low exam pass rates and progression to ST3 - Technical and non-technical skill development | Aims at individual trainee level |  |  |
| - Augment learning in theatre - Mastery of technical components of core procedures - Maintain technical skill proficiency - Safe physical and psychological space - Non-technical skills training |  | Perceived purpose of programmatic SBE |  |

| - Pockets of expertise and resource across Scotland - Non-technical skills programme - Experienced group of core faculty - Educational initiatives extensively developed |  | Pre-IST milestones in Core Surgical Training | COGNITIVE PARTICIPATION |
| --- | --- | --- | --- |
| - Adding to existing demands on trainers - Sustainability of funding - Changing persistent historic cultures |  | Predictable challenges to implementation |  |

| CODE | SUB-THEME | THEME | NPT CONSTRUCT |
| --- | --- | --- | --- |

| - Align with Government’s patient safety agenda - Clear communication with individual healthcare organisations - Fund resource for individual hospitals | Stakeholder engagement | Strategies for implementing programmatic SBE | COLLECTIVE ACTION |
| --- | --- | --- | --- |
| - Faculty development initiatives - Distributed leadership | Faculty involvement |  |  |
| - Establishing a core working group - Role and responsibilities of the Simulation Lead - Incorporate pockets of expertise - Map resource to curriculum | Processes for implementation |  |  |

| - Confidence in ability - Targeted feedback on performance - Ability to seek operative opportunities | Technical skills | Perceptions and experiences of programmatic SBE | REFLEXIVE MONITORING |
| --- | --- | --- | --- |
| - Healthy competition amongst peers - Peer and near-peer support and supervision - Collegiality amongst cohort - Individual goal-setting behaviours | Social learning environment |  |  |
| - Improved overall engagement - 100% recruitment into higher training - Improved curricular outcomes | Benefits to Core Surgical Training |  |  |
